# Innovative strategies for managing swine welfare during the COVID-19 pandemic in Iowa

**DOI:** 10.1093/tas/txab225

**Published:** 2021-12-09

**Authors:** Anna K Johnson, Chris J Rademacher, Jamee Eggers, Nicholas K Gabler, Laura L Greiner, Jeff Kaisand, Locke A Karriker, Suzanne T Millman, John F Patience, Brett C Ramirez, Lee L Schulz, Sherrie R Webb, Jason W Ross

**Affiliations:** 1 Department of Animal Science, Iowa State University, Ames, IA 50001, USA; 2 Iowa Pork Industry Center, Iowa State University, Ames, IA 50001, USA; 3 Department of Veterinary Diagnostic and Production Animal Medicine, Iowa State University, Ames, IA 50001, USA; 4 Iowa Pork Producers Association, Clive, IA 50325, USA; 5 Iowa Department of Agriculture and Land Stewardship, Des Moines, IA 50319, USA; 6 Swine Medicine Education Center, Iowa State University, Ames, IA 50001, USA; 7 Department of Biomedical Sciences, Iowa State University, Ames, IA 50001, USA; 8 Agricultural and Biosystems Engineering, Ames, IA 50001, USA; 9 Department of Economics, Iowa State University, Ames, IA 50001, USA; 10 American Association of Swine Veterinarians, Perry, IA 50220, USA

**Keywords:** animal care, coordination, mental-support, resources, tools

## Abstract

Coronavirus Disease 2019 (COVID-19) was declared a global pandemic on March 11, 2020 by the World Health Organization and its impact on animal agriculture in the United States was undeniable. By April, COVID-19 resulted in the simultaneous closure or reduced operations of many meat processing plants in the upper Midwest, leading to supply chain disruptions. In Iowa, the leading pork production and processing state, these disruptions caused producer uncertainty, confusion, and stress, including time-sensitive challenges for maintaining animal care. The Iowa Resource Coordination Center (IRCC) was quickly created and launched by the Iowa Department of Agriculture and Land Stewardship (IDALS). The IRCC included public representation from the Iowa Pork Producers Association (IPPA), Iowa Pork Industry Center (IPIC), and Iowa State University Extension and Outreach, and private partners including producers, veterinarians, and technical specialists. Supporting swine welfare, the IRCC provided information on management strategies, dietary alterations to slow pig growth, alternative markets, on-farm euthanasia, and mass depopulation under veterinary oversight. In a crisis, Iowa created a model that reacted to producers’ pragmatic, mental and emotional needs. This model could be quickly replicated with an introduction of foreign animal disease.

## UNITED STATES SWINE INDUSTRY

In 2020, the United States (U.S.) swine industry slaughtered approximately 131.6 M pigs producing 12.8 B kg of pork from around 6.3 M sows (USDA-NASS, 2020). Roughly 1 M pigs are transported daily (Roth and Spickler, 2014), and half are destined for harvest (USDA-AMS, 2021). To accommodate this daily harvest, the live animal supply chain functions with high efficiency, maximizing intensive animal housing infrastructure, based upon expected daily and weekly harvest quotas. Swine movements occur concomitantly with thousands of tons of feed from mills across the country, using ingredients sourced globally. Ingredient disruptions, labor issues, international policy, foreign animal diseases, and human pandemics alter animal and feed transport. COVID-19 created a massive labor disruption, resulting in packing plant closures and an overabundance of harvest-ready pigs. This article will describe how the Iowa swine industry responded to time-sensitive challenges to maintain swine welfare on-farm during a temporary disruption of the supply chain.

## COVID-19

COVID-19 is a respiratory disease caused by SARS-CoV-2, a new coronavirus discovered in 2019. The virus is thought to spread mainly from person to person through respiratory droplets produced when an infected person coughs, sneezes, or talks (CDC, 2021). On January 9, 2020, the World Health Organization (WHO) announced mysterious Coronavirus-related pneumonia in Wuhan, China. On January 21, the Centers for Disease Control confirmed the first U.S. Coronavirus case. On January 31, the WHO issued a global health emergency for just the sixth time, and on March 11, 2020 declared COVID-19 a pandemic (UN, 2021).

## MARKET DISRUPTION

Global markets were roiled by COVID‐19 with the swine and pork market being no exception. The market upheaval generated intense scrutiny among policy makers, the media, producers, and allied industry. Agricultural economists were asked to explain and monitor demand and supply disruptions, and price implications (Hart et al., 2020; ISU, 2020; Lusk et al., 2021). The pandemic consequences were difficult for swine producers and by October 24, 2020, the USDA Coronavirus Food Assistance Program (CFAP) payments totaled $622.79 M (CFAP 1) and $538.77 M respectively (CFAP 2; USDA, 2020a, 2020b).

## ANIMALS DURING TIMES OF CRISIS AND ONE WELFARE

Appleby and Stokes (2008) noted that a major reason to consider animals during a “*time of crisis*” (defined as a foreign animal disease, natural disaster, or human pandemic), is the huge scale of human-animal dependence. Humans rely on animals for transportation, social status, cultural identification, nutrition, and income. In turn, it is society’s responsibility to protect animals. “*Animal welfare means the physical and mental state of an animal in relation to the conditions in which it lives and dies. An animal experiences good welfare if the animal is healthy, comfortable, well nourished, safe, is not suffering from unpleasant states such as pain, fear and distress, and is able to express behaviours that are important for its physical and mental state. Good animal welfare requires disease prevention and appropriate veterinary care, shelter, management and nutrition, a stimulating and safe environment, humane handling and humane slaughter or killing*” (OIE, 2021). Animal welfare has been based on the Five Freedoms (freedom from hunger and thirst, discomfort, pain, injury or disease and fear and distress and the freedom to express normal behavior), and these have evolved into the Five Domains with a focus on positive affective states (nutrition, environment, physical health, behavior, and mental; Mellor et al., 2020). Building on the Domains, One Welfare is an emerging national, global, and holistic movement that recognizes that animal welfare, biodiversity, and the environment are connected to human well-being (Bourque, 2017; Garcia, 2017). The One Welfare platform helped formulate Iowa’s swine welfare, farmer mental health, and environmental conservation responses during COVID-19.

## IOWA RESOURCE COORDINATION CENTER

The formal opening of the Iowa Resource Coordination Center (IRCC) occurred on April 30, 2020. The IRCC was a private-public partnership between the Iowa Department of Agriculture and Land Stewardship (IDALS), Iowa State University (ISU), Iowa Pork Producers Association (IPPA), and producers, veterinarians, and technical specialists. The IRCC was designed to be a centralized point of all current information relevant to the pandemic-induced supply chain disruptions to ensure all farmers had access to the best resources, assistance, and technical information through personalized customer telephone or web service. The IRCC backbone was a formalized incident management structure, which followed a similar structure to what can be used during foreign animal disease outbreaks. To access all IRCC resources, visit https://www.ipic.iastate.edu/covid19.html. The COVID-19 pandemic impacted pig welfare on-farm due to overcrowding, which increased the risk of aggression, discomfort, and resource competition. Several solutions were deployed through the IRCC to address these swine welfare concerns, and a few notable examples are discussed next.

### Diets and Nutrition to Slow Growth

The aim was to deliberately reduce function by slowing growth, but doing it in a manner that would not cause pig hunger and competition. A rapid response resulted in the first set of data to be released to the swine industry in real-time that compared approaches to slow or stop growth while preserving swine welfare and pork carcass quality. Helm et al. (2021a) evaluated levels of neutral detergent fiber, amino acid reductions, and changing the dietary electrolyte balance through the addition of an acidogenic salt, anhydrous calcium chloride. Dietary calcium chloride (4% inclusion) slowed the growth of finishing pigs to approximately 7.5 kg, whereas reducing soybean meal and synthetic amino acids via the inclusion of up to 97% corn, resulted in 13.5 kg over the same 28 d trial. These University trials were validated commercially (Norton et al., 2020; Helm et al., 2021b; Rao et al., 2021). Webinars were held to update nutritionists and farmers on the results including information on the economic tradeoffs of feeding longer and switching to diets that may slow growth (Euken et al., 2020). In the first webinar, there were 264 individuals representing more than 50% of the U.S. sow ownership plus individuals from Canada. Extension publications, created to address questions, were downloaded over 1,400 times in the first 9 months (Gabler et al., 2020).

### Heavier Pig Prioritization

The aim was to deliberately market larger pigs to increase space allowance. This improved comfort, gave pigs control to eat and drink freely, hence reducing frustration. Due to supply chain disruptions, packing plants, buyers, and farmers cooperated to prioritize accepting heavier pigs first. Typically, overweight pigs have been financially penalized by packing plants. However, during COVID-19 the severity of overweight pigs being delivered was used as a proxy by packers to estimate which farmers had a more overcrowded supply of live pigs, which in turn enabled prioritization. Additionally, farmers pursued alternative marketing strategies including state-inspected and custom-exempt meat processing facilities, food bank donation programs, auction markets, and private sales. The Pass the Pork program gave Iowa pig farmers the ability to bring nearly 200,000 pork servings to food-insecure Iowans between April and July 2020. In June 2020, the Iowa State University Meats Laboratory joined the effort to help process pigs to support the Pass the Pork program.

### Euthanasia and Depopulation

Nutritional and other management strategies provided a temporary stopgap but did not entirely resolve the associated backlog of market-ready pigs. The aim of these efforts was to selectively euthanize pigs on-farm using approved euthanasia methods to improve the general pig population’s welfare. At the height of slaughter disruptions, it was estimated that 5 to 6 M head may have been euthanized between May and September 2020 (Meyer, 2020; Miller, 2020). Farmers, in conjunction with their veterinarians euthanized older parity sows, pigs housed in hospital pens, or pigs deemed as “*not thriving*.” Next, euthanasia decisions were made on healthy pigs to provide additional space. Once these selective euthanasia decisions had been exhausted, depopulation using approved methods or techniques permitted under exceptional circumstances were deployed. The American Association of Swine Veterinarians (AASV) adopted a position statement on strategies for responding to COVID-19 pandemic processing disruptions on May 19, 2020:

*Swine producers should work with their veterinarian to develop situation-specific strategies to deliver optimal care for pigs affected by the processing disruption due to the COVID-19 pandemic. In exercising their professional responsibilities, veterinarians should ensure:*

- *Actions are consistent with veterinary professional obligations, and conform to acceptable standards of veterinary practice and available scientific literature;*
- *Actions optimize the health, safety, and welfare outcomes for the animals and humans within the constraints of the COVID-19 pandemic; and*
- *Actions are consistent with applicable federal, state, and local regulations.*

*If depopulation must be considered, veterinarians should reference the AVMA guidelines for the Depopulation of Animals. Priority should be given to those classified as “Preferred” but the circumstances surrounding the COVID-19 processing disruption may require the use of methods classified as “Permitted in Constrained Circumstances.”*

The American Veterinary Medical Association (AVMA) has defined depopulation as the “rapid destruction of a population of animals in response to urgent circumstances with as much consideration given to the welfare of the animals as practicable” (AVMA, 2019). It should be noted that depopulation is different from euthanasia with different contexts and criteria. For each species, the AVMA Guidelines for the Depopulation of Animals classified methods of depopulation into one of three categories: preferred, permitted in constrained circumstances, and not recommended (Table 1; AVMA, 2019). The IRCC used the AVMA depopulation guidelines as their reference when aiding farmers with information and resources. Farmers faced with the prospect of having to depopulate pigs worked with their veterinarian to determine the most feasible approach to achieve this task by first considering all methods of depopulation classified as “Preferred” and only using methods classified as “Permitted in Constrained Circumstances” if none of the preferred methods were feasible. There were many factors that had to be considered when choosing a depopulation method including animal welfare, legal requirements, time constraints, animal ownership, personnel availability, worker physical and mental health and safety, operator and observer impact, public perception, animal environment, number and size of pigs, animal handling conditions, equipment or resource availability, biosecurity, carcass removal, and disposal. Each farm was unique in its resource availability and constraints, so conclusions on the depopulation method varied from farm-to-farm. The use of anesthetic overdose or injectable anesthetics and euthanasia agents pose significant limitations for carcass disposal in accordance with state regulations and, therefore, neither were considered suitable options for depopulation. Field reports on the veterinary use of sodium nitrite revealed inconsistent protocols and applications to achieve depopulation in the time targets, which makes this method highly impractical. Availability of carbon dioxide (CO_2_) to use for depopulation was highly dependent on geographical location. Most CO_2_ in the Midwest is derived from ethanol production, which was also experiencing production disruption due to COVID-19 (Baysinger et al., 2021; U.S. Energy Information Administration, 2021). Use of gunshot is limited to grow-finish and adult pigs, however, the quantity and type of ammunition needed for depopulation were limited in availability in many locations (Zent, 2020). Use of penetrating captive bolt is also a method recommended for grow-finish and adult pigs but adapting this method for depopulation of a large population of animals may be difficult for some farms because multiple devices are required to limit overheating, reduce cleaning demand, and prevent shooter fatigue. Individual restraint is not possible for rapid throughput of large populations so alternative restraint devices (e.g., single-file chutes, center-track restrainers, or v-track restrainers) are needed, which are not readily available on farms. This restraint limitation also applies to electrocution. Ventilation shutdown plus (VSD+) was used by a few farms when no other method was deemed feasible. Baysinger et al. (2021) published a methodology for VSD with the addition of supplemental heat and moisture that successfully achieved the AVMA requirement of at least 95% mortality within 1 h. This method required significant engineering, logistics, and process controls to be effective and is not a viable option for every farm. Field reports from state animal health officials suggest that widespread depopulation did not occur, which is supported by the increase in pigs passing through auction and specialty markets and the sustained increase in pig live weights arriving at packing plants once operation capacities resumed. The dietary adjustment strategies employed by farmers to slow pig growth and modifications made by slaughter facilities to ensure worker safety and continued operation were important contributing factors minimizing the need for depopulation.

**Table 1.** AVMA guidelines for the Depopulation of Animals: Chapter 4: Swine (2019)

Category		
Preferred	Permitted in constrained circumstance	Not recommended
Carbon dioxide	Sodium nitrite	None listed
Electrocution	Ventilation shutdown plus (VSD+)	
Gunshot	Compounded or nonpharmaceutical-grade injectable anesthetics and euthanasia agents	
Penetrating captive bolt		
Movement to slaughter		
Non-penetrating captive bolt		
Manual blunt force trauma		
Anesthetic overdose		

### Farmer Mental Support

Farmers raising food-producing animals have a strong identity attached to the nobility of feeding people essential nutrients while also providing for their immediate and future financial security. The massive overabundance of live pigs created a dual strain for farmers. Not only were pigs that had been well-cared for at significant risk of not being able to serve their food-producing purpose, but farmers were also experiencing financial calamity, as the over-supply of market pigs dramatically reduced live pig value. Farmers were faced with making impossible decisions related to euthanizing healthy pigs on-farm to create additional space or depopulation due to lack of a supply outlet. In normal day-to-day operations, farmers have reported experiencing stress when euthanizing pigs that they have been tasked with caring for (Yarian, 2021), defined as the “*caring-killing paradox*” (Arluke, 1994). Whiting and Marion (2011) have described the mass depopulation operational logistics of healthy, surplus market pigs in Canada due to an animal disease pandemic. These authors noted that participants who both individually and collectively had many years of slaughter and animal welfare disaster experience suffered significant mental health challenges, describing this as Perpetration-Induced Traumatic Stress. As a result, the Iowa Pork Industry Center (IPIC) hosted a webinar featuring Dr. D. Brown, a program specialist in Human Sciences Extension and Outreach with a specialization in behavioral health, and Dr. C. Schmitt of Pipestone Veterinary Services to help those affected by the supply chain disruptions recognize and respond to a deterioration in mental health. The AASV partnered with Dr. E. Strand, a licensed clinical social worker, resiliency coach, and founding director of Veterinary Social work, to begin offering HEARD VET in May 2020. This was a confidential, virtual swine veterinarian peer social support group for AASV members to share or listen to experiences unique to swine veterinarians.

## LESSONS LEARNED AND TAKE-HOME MESSAGE

The impacts of COVID-19 for Iowa and many parts of the U.S. swine industry were intense but luckily short (mid-March to early Fall 2020). While the swine industry has invested significant time and effort into planning and preparing its response to the possible introduction of foreign animal disease, the COVID-19 market disruption exposed depopulation resource limitations and knowledge gaps. Farmers, state and federal animal health officials did not have the resources readily available to depopulate large numbers of animals in an efficient and safe manner. Therefore, it is advised that the swine industry partners with state and federal government to ensure these resources are in place for use when future emergencies arise. Furthermore, several research projects to investigate depopulation strategies are ongoing. Success of the IRCC was the collaborations between multiple organizations (government, university, allied industry, and trade associations) that had overlapping stakeholders. This was essential for maximizing the likelihood of positive stakeholder outcomes. It was effectively accomplished by frequent joint communications through the IRCC leadership that focused on assimilating stakeholder input and disseminating impactful information. Several key resources were created such as webinars, peer-review publications, and extension articles that addressed packing plant utilization on livestock prices (Tonsor and Schulz, 2020a), meat availability (Tonsor et al., 2020), and pig inventories (Schulz, 2020a, 2020b, 2020c). Finally, pig welfare on-farm was forefront and center in every decision. The pandemic highlighted the importance of having access to local swine veterinarians and welfare experts to help educate farmers on key welfare decisions in real-time. Efforts included providing welfare material and tools, and helping support and guide euthanasia and depopulation decisions. In a crisis, Iowa created a model that reacted to swine welfare and the producers’ pragmatic, mental, and emotional needs. The take-home message of the IRCC efforts can be summarized as follows “*the ability to adapt and begin the process of recovery has been remarkable*” (Tonsor and Schulz, 2020b, p. 16).
